# Association between Lower-to-Upper Ratio of Appendicular Skeletal Muscle and Metabolic Syndrome

**DOI:** 10.3390/jcm11216309

**Published:** 2022-10-26

**Authors:** Hyun Eui Moon, Tae Sic Lee, Tae-Ha Chung

**Affiliations:** 1 Department of Family Medicine, Wonju Severance Christian Hospital, Yonsei University Wonju College of Medicine, Wonju 26426, Korea; 2 Research Group of Functional Medicine and Preclinical Disease, Wonju 26426, Korea; 3 Institute for Global Health Care and Development, Wonju 26426, Korea

**Keywords:** metabolic syndrome, sarcopenia, muscle mass ratio

## Abstract

(1) Background: Metabolic syndrome (MetS) is a cluster-based disorder comprising several pre-disease or pre-clinical statuses for diabetes, hypertension, dyslipidemia, cardiovascular risk, and mortality. Appendicular skeletal muscle (ASM), or lean mass, is considered the main site of insulin-mediated glucose utilization. Therefore, we aimed to reveal the association between lower appendicular skeletal muscle mass to upper appendicular skeletal muscle mass ratio (LUR) and risk for MetS. (2) Methods: We analyzed the 2008–2011 Korean National Health Examination and Nutrition Survey (KNHANES) data. Quintiles of lower ASM to upper ASM ratio (LUR) were categorized as follows: Q1: ≤2.65, Q2: 2.66–2.80, Q3: 2.81–2.94, Q4: 2.95–3.11, and Q5: ≥3.12 in men and Q1: ≤3.00, Q2: 3.01–3.18, Q3: 3.19–3.36, Q4: 3.37–3.60, and Q5: ≥3.61 in women. Multivariate logistic regression models were used after setting MetS and the LUR quintiles as the independent and dependent variables and adjusting for covariates. (3) Result: In men, MetS in accordance with the LUR quintiles exhibits a reverse J-curve. All groups from Q2 to Q5 had a lower odds ratio (OR) (95% CI) for MetS compared to the Q1 group. The lowest OR (95% CI) of 0.85 (0.80–0.91) was observed in Q4. However, in women, the figure shows a sine curve. Compared to the Q1 group, the Q2 and Q3 groups had a higher OR, while the Q4 and Q5 groups presented a lower OR. Among them, the OR (95% CI) in the Q4 group was lowest, at 0.83 (0.76–0.91). (4) Conclusions: While total appendicular skeletal muscle mass is important to prevent MetS, it is necessary to maintain an optimal ratio of muscle mass between the upper and lower appendicular skeletal muscle mass.

## 1. Introduction

Metabolic syndrome (MetS), also referred to as “insulin resistance syndrome” and “Syndrome X”, is a cluster-based disorder comprising several pre-disease or pre-clinical statuses for diabetes, hypertension, and dyslipidemia [1,2]. MetS is related to chronic diseases, such as type 2 diabetes (T2D), hypertension, and cardiovascular disease [3]. In men with MetS, cardiovascular disease and overall mortality are increased, even in the absence of baseline CVD and diabetes. Recently, Cho et al. [4] advocated to define pre-MetS as the presence of one or two components among five components of MetS; the pre-MetS phenotype is related to new-onset hypertension, T2D, and MetS.

Appendicular skeletal muscle (ASM), or lean mass, is considered the main site of insulin-mediated glucose utilization. Therefore, loss of ASM could drive metabolic impairments [5]. Cumulative clinical evidence supports the relations between age-related loss of muscle mass and function, called sarcopenia, and insulin resistance, mitochondrial dysfunction, and systemic inflammation [6,7]. Specifically, decreased lower extremity appendicular skeletal muscle (LASM) is related to high risk for obstructive coronary heart disease [8]. One study reported that low LASM mass is associated with an increased ratio of hospitalization in patients with type 2 diabetes [9]. Interestingly, this study showed that upper lean mass could not predict hospitalization. Another study reported that LASM mass is more highly associated with insulin resistance and cardiovascular risk than upper ASM mass [10].

Some studies have identified an association between body composition and chronic metabolic diseases [9,10]. However, these studies have several limitations, such as measuring muscle mass by bioelectrical impedance analysis or use of data with small groups. Furthermore, few studies have analyzed the lean muscle mass ratio at different body sites [11]. Muscle mass is influenced by many factors, such as weight, height, nutrition, physical activity, chronic diseases, and aging [12]. Thus, previous studies have focused on weight and height as modifiers, such as ASM/Weight or ASM/Height^2^. On the other hand, many studies have shown that lower extremity appendicular skeletal muscle is more important for preventing MetS. Therefore, we hypothesized that lower to upper appendicular skeletal muscle mass ratio is more useful in predicting MetS in a muscle-declining population.

## 2. Materials and Methods

### 2.1. Study Population

We analyzed data from the 2008–2011 Korean National Health Examination and Nutrition Survey (KNHANES), a cross-sectional study that is performed annually by the Korea Disease Control and Prevention Agency.

Sampling weights indicating the probability of selection were derived by three steps. The primary sample units (PSU) were chosen from a sampling frame consisting of all census blocks or resident registration addresses. Of all the dwelling units in a PSU, 20 households were selected for the on-site survey. Final selection occurred within a household, where all members older than one year were selected to participate. Thus, our results represent the whole landscape of the Korean population.

We enrolled those who were 40 years of age or older. The following subjects were excluded: those without complete data on medical history, lifestyle-related and anthropometric data, and laboratory markers and those with previously diagnosed cancer, chronic obstructive disease, myocardial infarction, angina, or chronic kidney disease.

Each subject in the present study provided informed consent. All analyses using the KNHANES data were performed according to the Declaration of Helsinki. Because the Institutional Review Board (IRB) of the Korea CDC (2008-04EXP-01-C, 2009-01CON-03-2C, 2010-02CON-21-C, 2011-02CON-06-C) approved the 2008–2011 KNHANES survey, additional IRB approval was not required for the present study.

### 2.2. Definition of Metabolic Syndrome (MetS)

MetS elements were defined according to the Joint Interim Statement of the International Diabetes Federation Task Force on Epidemiology and Prevention; National Heart, Lung, and Blood Institute; American Heart Association; World Heart Federation; International Atherosclerosis Society; and International Association for the Study of Obesity [2]. In detail, the component definitions were as follows: (1) waist circumference ≥ 90 cm in men and ≥85 cm in women [13]; (2) serum triglycerides ≥ 150 mg/dL or drug treatment for elevated triglycerides (TG); (3) serum HDL-cholesterol < 40 mg/dL in men and <50 mg/dL in women, or drug treatment to reduce HDL-cholesterol; (4) systolic blood pressure (SBP) ≥ 130 mmHg and diastolic blood pressure (DBP) ≥ 85 mmHg, or use of antihypertensive medications; and (5) fasting blood glucose ≥ 100 mg/dL or use of antidiabetic medications. We defined subjects with three or more of the above components as having MetS.

### 2.3. Measurement of Body Composition

In the KNHANES, body composition was measured using DXA (Dual X-ray Absorptiometry; QDR 4500A; Hologic Inc., Bedford, MA, USA). The technicians performed the DXA measurements using a previously described protocol [14]. The coefficients of variation were 1.9% for the lumbar spine, 2.5% for the femoral neck, and 1.8% for the total femur [14]. Appendicular skeletal muscle mass (ASM) was calculated as the sum of the lean mass of the four limbs. The LUR was defined as the lower appendicular skeletal muscle mass divided by the upper appendicular skeletal muscle mass.

### 2.4. Measurements of Variables

Data on the participants’ medical conditions were obtained through face-to-face interviews and questionnaires. The participants answered questions about their chronic diseases and health-related behaviors, such as smoking, alcohol use, and exercise. A smoker was defined as a participant with a current smoking status. The participants were asked to report their average alcohol intake per week. We defined heavy alcohol consumption as at least 140 g per week for men and 70 g per week for women. Exercise was measured using the International Physical Activity Questionnaire (IPAQ), which categorizes low, moderate, and vigorous physical activity. We defined appropriate physical activity as moderate to vigorous activity more than 3 days per week. Waist circumference was measured with the participant wearing lightweight clothes. Blood pressure was measured using an appropriately sized arm cuff and a mercury sphygmomanometer after resting for at least 5 min in a seated chair and was measured 3 times by medical staff; the average of the second and third values was used. Participants underwent blood collection after at least 8 h of fasting. The samples were analyzed within 24 h. KNHANES-associated blood analysis was performed using a model 7600 auto-analyzer (Hitachi, Tokyo, Japan) for serum fasting glucose, creatinine, and lipid profiles. An XE-2100 (Sysmex, Tokyo, Japan) was used to analyze white blood cell count.

### 2.5. Statistics

All data in the present study were described as mean ± standard error for continuous variables and as number and ratio (%) for categorical variables. Korean men and women were separately categorized into LUR quintile groups as follows: Q1: ≤2.65, Q2: 2.66–2.80, Q3: 2.81–2.94, Q4: 2.95–3.11, and Q5: ≥3.12 in men and Q1: ≤3.00, Q2: 3.01–3.18, Q3: 3.19–3.36, Q4: 3.37–3.60, and Q5: ≥3.61 in women. For comparative analyses of continuous variables based on the linear trends of the LUR quintiles, we used a one-way analysis of variance after setting the average values of each quintile group as continuous variables. A chi-square test was implemented for differential analyses of categorical variables based on the LUR quintiles.

The weighted sample was used in logistic regression (LR) analyses. Sample weights assigned to the subjects by the data constructors were adopted to reflect the whole Korean population. Basically, multivariate LR models were used after setting MetS and LUR (continuous or categorical group) as the independent and dependent variables, respectively. All statistical analyses were conducted using the R program (version 4.1.3). We considered a *p*-value less than 0.05 as significant.

## 3. Results

Table 1 shows the baseline characteristics according to the LUR quintiles in Korean men. In the stepwise transition from the first to the fifth quintiles, the following variables exhibited a positive trend in men: age, total ASM, LASM, BMI, waist circumference, serum creatinine, fasting glucose, and percentage of diagnosed MetS according to previously described criteria. Conversely, the following variables showed a negative trend: upper ASM, smoking, and HDL. In Korean women, baseline characteristics according to the LUR quintiles are summarized in Table 2. The following variables showed a positive trend from the first to the fifth quintiles in women: total ASM, LASM, exercise, BMI, and waist circumference. Conversely, those that showed a negative trend were upper ASM, age, percentage of MetS, menopause, systolic blood pressure, diastolic blood pressure, fasting glucose, HDL, TG, and WBC count.

Table 3 describes the ORs for MetS across the LUR quintiles, as calculated by multiple logistic regression. After adjusting for age, smoking, alcohol, exercise, BMI, ASM, WBC, and serum creatinine in men, the lowest OR was exhibited in the Q4 group. After adjustment, the Q4 group also had the lowest OR in women.

**Figure 1:**
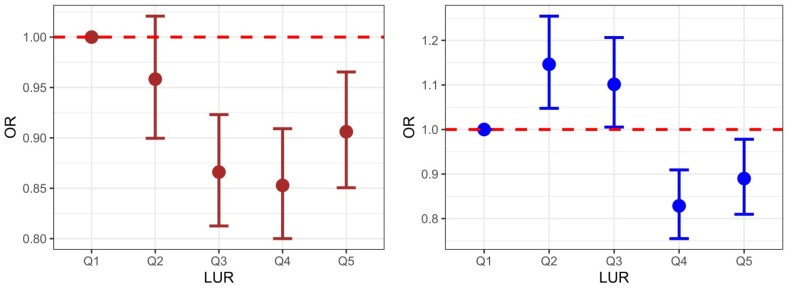
Odds ratio and confidence interval for MetS across the LUR quintiles in men and women, after adjusting for age, smoking, exercise, alcohol, ASM, BMI, WBC, creatinine, and menopause (women). Abbreviations: ASM, appendicular skeletal muscle; BMI, body mass index; WBC, white blood count.

Figure 1 presents the OR and CI for each quintile in men and women. The odds ratio was calculated after adjusting for age, smoking, exercise, alcohol, BMI, ASM, WBC, creatinine, and menopause (in women). The figure shows a reverse J-curve for men. All groups from Q2 to Q5 had a lower OR (95% CI) for MetS compared to Q1. The lowest OR (95% CI) in the Q4 group, which was 0.853 (0.800–0.909), was lower than the ORs in Q3 (0.866, 0.813–0.923) and Q5 (0.906, 0.850–0.965). 

In contrast to the reverse J-curve seen in men, the figure for women shows a sine curve. Compared to the Q1 group, Q2 and Q3 had a higher OR (95%), while Q4 and Q5 presented a lower OR (95%). Among them, the OR (95% CI) in the Q4 group was the lowest, at 0.829 (0.755–0.909).

## 4. Discussion

Our study showed that, in Korean men and women, the LASM mass to upper ASM mass ratio has a reverse J-curve and a sine curve, respectively, in relation to the risk of MetS. This suggests that the optimal LUR for decreasing the prevalence of MetS is 2.95–3.11 in men and 3.37–3.60 in women.

Several previous studies have shown relationships between body composition and various metabolic diseases, such as hypertension, diabetes, cardiovascular disease, and MetS [10,15,16]. Some studies showed that hip circumference or lower muscle mass is important for predicting MetS and mortality [15,17]. In another study, in type 2 diabetes patients, lower limb skeletal muscle is significantly correlated with insulin resistance and an increasing number of cardiovascular diseases [10]. Heo et al. reported that thigh muscle mass is inversely related to insulin resistance in men, particularly in those with higher BMI [18]. In addition, previous studies have shown that insulin resistance and chronic subclinical inflammation are closely related to MetS [10,19]. 

Although the exact mechanism is unknown, several plausible processes can explain the relationship between body composition and MetS. Skeletal muscle is considered an endocrine organ because it releases myokines that mediate crosstalk in autocrine and paracrine manners between muscle, adipose tissue, liver, brain, and other organs [5]. Therefore, skeletal muscle is considered a critical site for insulin-mediated glucose utilization, which may explain why the loss of skeletal muscle mass can lead to MetS. Moreover, skeletal muscle, MetS, and inflammation are closely related. Blood leukocytes are an inexpensive and widely utilized marker of inflammation. Previous studies have shown that increased total leukocytes, neutrophils, and lymphocytes have a positive relationship with MetS [20]. Even if the values are in the normal range, higher leukocyte count is associated with type 2 diabetes, cardiovascular disease, and MetS [20,21]. Chronic low-grade inflammation, called subclinical inflammation, plays an important role in decreasing skeletal muscle and positively affects waist circumference, fasting plasma glucose, HOMA-IR, and triglyceride levels [22,23]. Finally, total ASM might be essential for insulin metabolism and chronic subclinical inflammation. Consistent with the results of previous studies, our study confirmed that the Q4 group had the lowest WBC count in both sexes, and the lowest fasting glucose in women. 

One of the main causes of MetS is increased insulin resistance. Insulin resistance causes compensatory hyperinsulinemia, which induces glycogenesis, protein synthesis reduction, and accelerated protein degradation [24]. Skeletal muscle accounts for 80% of glucose clearance by glucose transporter 4, and insulin resistance due to reduced skeletal muscle mass causes lipolysis, releases free fatty acids from adipose tissue, and inhibits the growth hormone-insulin-like growth factor 1 axis. In addition, myofibers in skeletal muscle can secrete proteins and myokines, which enhance metabolic abnormalities [25]. Thus, aging or underlying disease-related skeletal muscle loss can exacerbate abnormalities in glucose metabolism. Insulin resistance results in increased glycogenesis, expression of sterol regulatory-element bind protein 1c (SREBP-1c), supply of free fatty acids, inhibition of beta-oxidation, and altered triglyceride transport, causing the accumulation of triglycerides in skeletal muscle and liver [26]. Furthermore, patients with increased insulin resistance have a higher risk for loss of lean body mass and worse glycemic control correlated with decreased physical performance [27]. Low lean body mass is responsible for increasing the risk of diabetes and insulin resistance. Diabetes also has a bidirectional relationship with lean body mass loss through low-grade systemic inflammation, which can be marked by interleukin-6 (IL-6), tumor necrosis factor-alpha (TNF-alpha), C-reactive protein (CRP), and white blood cell count [20,28].

This study has several strengths. Generally, LASM mass is higher than upper ASM mass. However, in this study, we hypothesized an optimal ratio for lower to upper appendicular skeletal muscle mass. The ORs for MetS across the LUR quintiles show a reverse J-curve in men and a sine curve in women. According to these results, maintaining a specific ratio of lower to upper muscle mass might be necessary for preventing MetS.

In addition, within our knowledge, a single study had investigated the relationship between upper to lower appendicular skeletal muscle ratio and MetS risk. A recent study analyzing the KNHANES data reported that the arm-to-leg and limbs-to-trunk body fat-free mass ratios exhibited a negative relationship with the prevalence of MetS, which was inconsistent with the results obtained in our study [11]. That study reported a higher upper-extremity-to-lower-extremity ASM mass ratio, called arm-to-leg ratio, indicating a lower prevalence of MetS. The ORs of the arm-to-leg fat-free mass ratio in relation to MetS were 0.52 in men and 0.61 in women. Interestingly, their analyses highlighted the importance of upper extremity muscle mass in preventing MetS. In contrast, our study showed the importance of lower extremity muscle mass. This inconsistency might be caused by several study differences. For instance, their study only considered age, energy intake, water intake, smoking, alcohol drinking, physical activity, education, income, and survey year as covariates. On the other hand, our study pinpointed BMI, ASM, WBC, and serum creatinine, which are closely related to MetS. Although MetS does not have diagnostic criteria based on BMI, it has long been known as a major risk factor for MetS. WBC count and serum creatinine, previously described as subclinical inflammatory markers, have a relationship with insulin resistance, waist circumference, fasting plasma glucose, and triglyceride levels, which are risk factors of MetS [20,29]. Furthermore, our results demonstrated that the higher LUR quintile group showed a higher WBC count and a higher serum creatinine level. In women, in the stepwise transition from the Q1 to Q5 groups, waist circumference, triglyceride level, and fasting plasma glucose all decreased, which also supported our findings.

The number of groups is also a strength of our study. We divided the total sample into five groups instead of four, as in the previous study. Because our study revealed that the Q4 group showed a lower OR than the Q3 and Q5 groups, a smaller number of groups could mask this unique relationship between LUR and MetS.

Our study showed that the LUR Q4 group, and not the Q5 group, had the lowest OR for MetS in men and women. On the other hand, in both men and women, total ASM was highest in the Q5 group. This suggests not only that MetS is related to total muscle mass but also that there is an optimal ratio between lower ASM and upper ASM. Moreover, the trends vary between men and women. This difference could be affected by the sex hormone estrogen, which has been found to provide protective effects against adipocyte inflammation, oxidative stress, insulin resistance, and mitochondrial function [30,31]. Thus, the inconsistent results between the sexes could arise from sex hormone or reproductive history. Moreover, body fat composition may impact the results. Previous studies have shown a negative relationship between thigh circumference and MetS in men and women [16]. However, thigh circumference is mainly influenced by muscle in men but by fat in women [32]. This suggests that the relatively low LUR in women is more influenced by fat mass than muscle mass.

There are some limitations to the present study that should be considered when interpreting its findings. First, this is a cross-sectional study; thus, a causal relationship between LUR and MetS could not be established. Although a significant relationship between LUR and MetS is shown in the present study, it cannot be concluded whether an optimal LUR is a relieving factor actively involved in the development of MetS or just an epiphenomenon. Second, we excluded several diseases, including cancer, chronic obstructive pulmonary disease, myocardial infarction, angina, and chronic kidney disease, because they could induce cachexia [12], which influences muscle mass. We collected the data using interviews, which could be influenced by recall bias. Since this study used secondary data derived from the KNHANES, we could not collect data on sex hormone prescriptions, such as estrogen or testosterone; inflammatory markers, such as CRP or IL-6; or insulin resistance markers, such as HOMA-IR. Finally, although our findings may be generalizable because the KNHANES represents a diverse and large group, it is based on Korea’s demographic data. Thus, most of the participants were Asian, which requires further investigation to generalize the findings to other cultural groups.

## 5. Conclusions

We confirmed that the association between LUR and MetS is a reverse J-curve in men and a sine curve in women. Low ASM might be regarded as a risk for MetS in the muscle-declining population. However, although total ASM mass is important to prevent MetS, it is necessary to maintain an optimal ratio of muscle mass between the upper and lower ASM mass.

## Figures and Tables

**Table 1:** General characteristics according to the LUR quintiles in Korean men ^a^.

	LUR Quintile in Men					*p* for Trend ^b^
	Q1 (≤2.65)	Q2 (2.66–2.80)	Q3 (2.81–2.94)	Q4 (2.95–3.11)	Q5 (≥3.12)	
*n*	918	918	918	918	919	
Age, year	55.2 ± 0.34	56.9 ± 0.37	56.9 ± 0.37	57.2 ± 0.38	57.2 ± 0.38	<0.001
ASM, g	21,334.6 ± 108.69	21,335.7 ± 105.78	21,495.5 ± 103.16	21,490.3 ± 103.57	21,854.8 ± 102.56	<0.001
Upper ASM, g	6081.3 ± 31.31	5713.4 ± 28.42	5541.1 ± 26.67	5333.2 ± 25.69	5089.1 ± 24.56	<0.001
Lower ASM, g	15,253.3 ± 78.73	15,622.3 ± 77.5	15,954.4 ± 76.59	16,157.1 ± 78	16,765.7 ± 79.06	<0.001
LUR	2.5115 ± 0.0044	2.7348 ± 0.0016	2.8797 ± 0.0013	3.0298 ± 0.0016	3.3003 ± 0.0055	<0.001
MetS, *n*	313 (34.1)	340 (37)	331 (36.1)	348 (37.9)	375 (40.8)	0.047
Smoking, *n*	633 (69)	600 (65.4)	582 (63.4)	555 (60.5)	524 (57)	<0.001
Alcohol consumption, *n*	328 (35.7)	329 (35.8)	323 (35.2)	297 (32.4)	299 (32.5)	0.295
Exercise, *n*	352 (38.3)	313 (34.1)	321 (35)	295 (32.1)	324 (35.3)	0.085
BMI, kg/m^2^	23.5 ± 0.1	23.8 ± 0.1	23.9 ± 0.09	24.1 ± 0.1	24.6 ± 0.09	<0.001
WC, cm	82.8 ± 0.31	84.7 ± 0.28	85.3 ± 0.26	85.9 ± 0.27	87.2 ± 0.26	<0.001
Systolic BP, mmHg	125.3 ± 0.57	125.3 ± 0.56	125 ± 0.54	124.6 ± 0.54	124.8 ± 0.56	0.313
Diastolic BP, mmHg	81.4 ± 0.36	80.6 ± 0.37	80.4 ± 0.35	80.3 ± 0.35	80.8 ± 0.35	0.187
Fasting glucose, mg/dL	102 ± 0.83	102.7 ± 0.9	103.4 ± 0.81	104.6 ± 0.99	104.2 ± 0.86	0.029
HDL, mg/dL	46.6 ± 0.39	45.7 ± 0.36	45.8 ± 0.37	44.6 ± 0.35	45.1 ± 0.37	0.001
TG, mg/dL	161.6 ± 5.35	172 ± 4.91	165.3 ± 4.54	168.1 ± 4.1	167.5 ± 4.67	0.555
Cr, mg/dL	0.939 ± 0.0063	0.9464 ± 0.0052	0.9441 ± 0.0066	0.9561 ± 0.0054	0.9742 ± 0.0052	<0.001
WBC count, n/μL	6535 ± 60.5	6500 ± 56.5	6438 ± 58.7	6413 ± 56.4	6484 ± 55.7	0.364

^a^ Continuous and categorical variables are described as mean ± standard error and frequency (percent). ^b^ *p* for trend was calculated using ANOVA test or chi-square test. Abbreviations: ASM, appendicular skeletal muscle; LUR, upper appendicular skeletal muscle mass ratio; MetS, metabolic syndrome; BMI, body mass index; WC, waist circumference; TG, triglycerides; HDL, high density lipoprotein; Cr, creatinine; WBC, white blood count.

**Table 2:** General characteristics according to the LUR quintiles in Korean women ^a^.

	LUR Quintile in Women					*p* for Trend ^b^
	Q1 (≤3.00)	Q2 (3.01–3.18)	Q3 (3.19–3.36)	Q4 (3.37–3.60)	Q5 (≥3.61)	
*n*	1212	1212	1212	1212	1213	
Age, year	61.9 ± 0.33	59.3 ± 0.33	57 ± 0.32	54.9 ± 0.31	52.7 ± 0.29	<0.001
ASM, g	13,786.2 ± 59.08	14,224.7 ± 59.45	14,379.2 ± 58.55	14,553.6 ± 59.9	14,648.8 ± 57.4	<0.001
Upper ASM, g	3612.5 ± 15.36	3474 ± 14.58	3361.8 ± 13.69	3247.1 ± 13.41	3028.3 ± 12.27	<0.001
Lower ASM, g	10,173.7 ± 44.49	10,750.7 ± 44.94	11,017.4 ± 44.93	11,306.5 ± 46.58	11,620.5 ± 45.8	<0.001
LUR	2.8189 ± 0.0043	3.0954 ± 0.0015	3.2776 ± 0.0015	3.4828 ± 0.0019	3.8447 ± 0.0059	<0.001
MetS, *n*	464 (38.3)	467 (38.5)	433 (35.7)	377 (31.1)	341 (28.1)	<0.001
Smoking, *n*	94 (7.8)	97 (8)	82 (6.8)	69 (5.7)	71 (5.9)	0.073
Alcohol consumption, *n*	64 (5.3)	99 (8.2)	83 (6.8)	79 (6.5)	85 (7)	0.082
Exercise, *n*	354 (29.2)	399 (32.9)	375 (30.9)	439 (36.2)	422 (34.8)	0.001
Menopause, *n*	888 (73.3)	803 (66.3)	737 (60.8)	641 (52.9)	585 (48.2)	<0.001
BMI, kg/m^2^	23.4 ± 0.1	23.9 ± 0.09	24 ± 0.09	24.2 ± 0.09	24.3 ± 0.09	<0.001
WC, cm	79.6 ± 0.28	81 ± 0.26	81.1 ± 0.26	81.4 ± 0.26	81.1 ± 0.27	<0.001
Systolic BP, mmHg	127.5 ± 0.54	125.9 ± 0.54	122.7 ± 0.5	120.6 ± 0.52	117.4 ± 0.48	<0.001
Diastolic BP, mmHg	77.5 ± 0.3	77.4 ± 0.29	77 ± 0.29	76.2 ± 0.3	75.8 ± 0.29	<0.001
Fasting glucose, mg/dL	99.3 ± 0.64	100.3 ± 0.71	99.4 ± 0.7	98.9 ± 0.67	96.3 ± 0.52	<0.001
HDL, mg/dL	48.7 ± 0.31	48.7 ± 0.33	49 ± 0.31	49.4 ± 0.32	50.2 ± 0.32	<0.001
TG, mg/dL	135.4 ± 2.74	131.2 ± 2.38	128.4 ± 2.49	122.2 ± 2.25	122.1 ± 2.21	<0.001
Cr, mg/dL	0.7028 ± 0.0035	0.714 ± 0.004	0.7136 ± 0.0034	0.7104 ± 0.0034	0.7093 ± 0.0034	0.413
WBC count, n/μL	5838 ± 45	5781 ± 46.1	5730 ± 44.8	5619 ± 46	5593 ± 43.9	<0.001

^a^ Continuous and categorical variables are described as mean ± standard error and frequency (percent). ^b^ *p* for trend was calculated using ANOVA test or chi-square test. Abbreviations: ASM, appendicular skeletal muscle; LUR, upper appendicular skeletal muscle mass ratio; MetS, metabolic syndrome; BMI, body mass index; WC, waist circumference; TG, triglycerides; HDL, high density lipoprotein; Cr, creatinine; WBC, white blood count.

**Table 3:** Odds ratio for MetS across the LUR quintiles in men and women.

Men	Q1 (<2.65)	Q2 (2.66–2.80)	Q3 (2.81–2.94)	Q4 (2.95–3.11)	Q5 (≥3.12)
Model 1 ^a^	1 (reference)	1.063 (1.016–1.112)	0.917 (0.877–0.96)	0.991 (0.947–1.037)	1.266 (1.213–1.321)
Model 2 ^b^	1 (reference)	1.046 (1.002–1.091)	0.978 (0.937–1.021)	1.028 (0.985–1.073)	1.282 (1.229–1.338)
Model 3 ^c^	1 (reference)	0.958 (0.899–1.021)	0.866 (0.813–0.923)	0.853 (0.800–0.909)	0.906 (0.850–0.965)
Women	Q1 (<3.00)	Q2 (3.01–3.18)	Q3 (3.19–3.36)	Q4 (3.37–3.60)	Q5 (≥3.61)
Model 1 ^a^	1 (reference)	1.278 (1.220–1.339)	1.386 (1.323–1.452)	1.155 (1.101–1.211)	1.248 (1.189–1.309)
Model 2 ^b^	1 (reference)	1.285 (1.226–1.346)	1.389 (1.326–1.456)	1.158 (1.104–1.214)	1.253 (1.194–1.314)
Model 3 ^d^	1 (reference)	1.146 (1.048–1.254)	1.101 (1.005–1.206)	0.829 (0.755–0.909)	0.890 (0.810–0.978)

^a^ Model 1 was calculated after adjusting for age. ^b^ Model 2 was calculated after adjusting for Model 1 + smoking, exercise, and alcohol. ^c^ Model 3 was calculated after adjusting for Model 2 + ASM, BMI, WBC, and creatinine. ^d^ Model 3 was calculated after adjusting for Model 2 + ASM, BMI, WBC, creatinine, and menopause.

## Data Availability

Not applicable.

## References

[1] Rochlani Y., Pothineni N.V., Kovelamudi S., Mehta J.L. (2017). Metabolic syndrome: Pathophysiology, management, and modulation by natural compounds. Ther. Adv. Cardiovasc. Dis..

[2] Alberti K.G.M.M., Eckel R.H., Grundy S.M., Zimmet P.Z., Cleeman J.I., Donato K.A., Fruchart J.C., James W.P.T., Loria C.M., Smith S.C. (2009). Harmonizing the metabolic syndrome: A joint interim statement of the international diabetes federation task force on epidemiology and prevention; National heart, lung, and blood institute; American heart association; World heart federation; International Atherosclerosis Society; and International Association for the Study of Obesity. Circulation.

[3] Lakka H.-M., Laaksonen D.E., Lakka T., Niskanen L.K., Kumpusalo E., Tuomilehto J., Salonen J.T. (2002). The Metabolic Syndrome and Total and Cardiovascular Disease Mortality in Middle-Aged Men. JAMA.

[4] Cho A.-R., Kwon Y.-J., Kim J.-K. (2021). Pre-Metabolic Syndrome and Incidence of Type 2 Diabetes and Hypertension: From the Korean Genome and Epidemiology Study. J. Pers. Med..

[5] DeFronzo R.A., Tripathy D. (2009). Skeletal Muscle Insulin Resistance Is the Primary Defect in Type 2 Diabetes. Diabetes Care.

[6] Kim T.N., Choi K.M. (2015). The Implications of Sarcopenia and Sarcopenic Obesity on Cardiometabolic Disease. J. Cell. Biochem..

[7] Marzetti E., Calvani R., Cesari M., Buford T.W., Lorenzi M., Behnke B.J., Leeuwenburgh C. (2013). Mitochondrial dysfunction and sarcopenia of aging: From signaling pathways to clinical trials. Int. J. Biochem. Cell Biol..

[8] Tibuakuu M., Zhao D., Saxena A., Brown T.T., Jacobson L.P., Palella F.J., Witt M.D., Koletar S.L., Margolick J.B., Guallar E. (2018). Low thigh muscle mass is associated with coronary artery stenosis among HIV-infected and HIV-uninfected men: The Multicenter AIDS Cohort Study (MACS). J. Cardiovasc. Comput. Tomogr..

[9] Hamasaki H. (2017). Lower Extremity Skeletal Muscle Mass, but Not Upper Extremity Skeletal Muscle Mass, Is Inversely Associated with Hospitalization in Patients with Type 2 Diabetes. J. Diabetes Res..

[10] Tajiri Y., Kato T., Nakayama H., Yamada K. (2010). Reduction of Skeletal Muscle, Especially in Lower Limbs, in Japanese Type 2 Diabetic Patients with Insulin Resistance and Cardiovascular Risk Factors. Metab. Syndr. Relat. Disord..

[11] Jung S., Park J., Seo Y.-G. (2021). Relationship between arm-to-leg and limbs-to-trunk body composition ratio and cardiovascular disease risk factors. Sci. Rep..

[12] Drescher C., Konishi M., Ebner N., Springer J. (2015). Loss of muscle mass: Current developments in cachexia and sarcopenia focused on biomarkers and treat-ment. J. Cachexia Sarcopenia Muscle.

[13] Kim B.Y., Kang S.M., Kang J.H., Kang S.Y., Kim K.K., Kim K.B., Kim B., Kim S.J., Kim Y.H., Kim J.H. (2021). 2020 Korean Society for the Study of Obesity Guidelines for the Management of Obesity in Korea. J. Obes. Metab. Syndr..

[14] Lim Y., Chun S., Lee J.H., Baek K.H., Lee W.K., Yim H.-W., Kang M.-I. (2016). Association of bone mineral density and diabetic retinopathy in diabetic subjects: The 2008–2011 Korea National Health and Nutrition Examination Survey. Osteoporos. Int..

[15] Cameron A.J., Magliano D., Shaw J.E., Zimmet P., Carstensen B., Alberti K.G.M., Tuomilehto J., Barr E.L.M., Pauvaday V.K., Kowlessur S. (2012). The influence of hip circumference on the relationship between abdominal obesity and mortality. Int. J. Epidemiol..

[16] Londoño F.J., Calderón J.C., Gallo J. (2012). Association between Thigh Muscle Development and the Metabolic Syndrome in Adults. Ann. Nutr. Metab..

[17] Moon J.H., Choo S.R., Kim J.S. (2015). Relationship between Low Muscle Mass and Metabolic Syndrome in Elderly People with Normal Body Mass Index. J. Bone Metab..

[18] Heo J.E., Shim J.-S., Lee H., Kim H.C. (2020). Association between the Thigh Muscle and Insulin Resistance According to Body Mass Index in Middle-Aged Korean Adults. Diabetes Metab. J..

[19] Roberts C.K., Hevener A.L., Barnard R.J. (2013). Metabolic Syndrome and Insulin Resistance: Underlying Causes and Modification by Exercise Training. Compr. Physiol..

[20] Jung C.-H., Lee W.-Y., Kim B.-Y., Park S.E., Rhee E.-J., Park C.-Y., Oh K.-W., Mok J.-O., Kim C.-H., Park S.-W. (2013). The Risk of Metabolic Syndrome According to the White Blood Cell Count in Apparently Healthy Korean Adults. Yonsei Med. J..

[21] Welsh C., Welsh P., Mark P.B., Celis-Morales C.A., Lewsey J., Gray S.R., Lyall D.M., Iliodromiti S., Gill J.M., Pell J. (2018). Association of Total and Differential Leukocyte Counts with Cardiovascular Disease and Mortality in the UK Biobank. Arter. Thromb. Vasc. Biol..

[22] Dalle S., Rossmeislova L., Koppo K. (2017). The Role of Inflammation in Age-Related Sarcopenia. Front. Physiol..

[23] Chung T.-H., Shim J.-Y., Lee Y.-J. (2015). Association between leukocyte count and sarcopenia in postmenopausal women: The Korean National Health and Nutrition Examination Survey. Maturitas.

[24] Bonaldo P., Sandri M. (2013). Cellular and molecular mechanisms of muscle atrophy. Dis. Model. Mech..

[25] Walsh K. (2009). Adipokines, Myokines and Cardiovascular Disease. Circ. J..

[26] Yoon J.W., Ha Y.-C., Kim K.M., Moon J.H., Choi S.H., Lim S., Park Y.J., Lim J.-Y., Kim K.W., Park K.S. (2016). Hyperglycemia is associated with impaired muscle quality in older men with diabetes: The korean longitudinal study on health and aging. Diabetes Metab. J..

[27] Nishikawa H., Asai A., Fukunishi S., Nishiguchi S., Higuchi K. (2021). Metabolic Syndrome and Sarcopenia. Nutrients.

[28] Kim T.N., Park M.S., Yang S.J., Yoo H.J., Kang H.J., Song W., Seo J.A., Kim S.G., Kim N.H., Baik S.H. (2010). Prevalence and determinant factors of sarcopenia in patients with type 2 diabetes: The Korean Sarcopenic Obesity Study (KSOS). Diabetes Care.

[29] Wang J., Li X., Han X., Yang K., Liu B., Li Y., Wu P., Liu X., Yu K., Dai X. (2015). Serum creatinine levels and risk of metabolic syndrome in a middle-aged and older Chinese population. Clin. Chim. Acta.

[30] Gupte A.A., Pownall H.J., Hamilton D.J. (2015). Estrogen: An Emerging Regulator of Insulin Action and Mitochondrial Function. J. Diabetes Res..

[31] White R.E., Gerrity R., Barman S.A., Han G. (2010). Estrogen and oxidative stress: A novel mechanism that may increase the risk for cardiovascular disease in women. Steroids.

[32] Kasai T., Ishiguro N., Matsui Y., Harada A., Takemura M., Yuki A., Kato Y., Otsuka R., Ando F., Shimokata H. (2015). Sex- and age-related differences in mid-thigh composition and muscle quality determined by computed to-mography in middle-aged and elderly Japanese. Geriatr. Gerontol. Int..

